# Adaptive evolution of *Methylotuvimicrobium alcaliphilum* to grow in the presence of rhamnolipids improves fatty acid and rhamnolipid production from CH_4_

**DOI:** 10.1093/jimb/kuac002

**Published:** 2022-02-03

**Authors:** Deepika Awasthi, Yung-Hsu Tang, Bashar Amer, Edward E K Baidoo, Jennifer Gin, Yan Chen, Christopher J Petzold, Marina Kalyuzhnaya, Steven W Singer

**Affiliations:** Biological Systems and Engineering Division, Lawrence Berkeley National Laboratory, Berkeley, CA 94720, USA; Biological Systems and Engineering Division, Lawrence Berkeley National Laboratory, Berkeley, CA 94720, USA; Biological Systems and Engineering Division, Lawrence Berkeley National Laboratory, Berkeley, CA 94720, USA; Biological Systems and Engineering Division, Lawrence Berkeley National Laboratory, Berkeley, CA 94720, USA; Biological Systems and Engineering Division, Lawrence Berkeley National Laboratory, Berkeley, CA 94720, USA; Biological Systems and Engineering Division, Lawrence Berkeley National Laboratory, Berkeley, CA 94720, USA; Biological Systems and Engineering Division, Lawrence Berkeley National Laboratory, Berkeley, CA 94720, USA; Department of Biology, San Diego State University, San Diego, CA 92182, USA; Biological Systems and Engineering Division, Lawrence Berkeley National Laboratory, Berkeley, CA 94720, USA

**Keywords:** Methanotrophs, Methane, Rhamnolipids, Adaptive lab evolution, Fatty acid secretion

## Abstract

Rhamnolipids (RLs) are well-studied biosurfactants naturally produced by pathogenic strains of *Pseudomonas aeruginosa.* Current methods to produce RLs in native and heterologous hosts have focused on carbohydrates as production substrate; however, methane (CH_4_) provides an intriguing alternative as a substrate for RL production because it is low cost and may mitigate greenhouse gas emissions. Here, we demonstrate RL production from CH_4_ by *Methylotuvimicrobium alcaliphilum* DSM19304. RLs are inhibitory to *M. alcaliphilum* growth (<0.05 g/l). Adaptive laboratory evolution was performed by growing *M. alcaliphilum* in increasing concentrations of RLs, producing a strain that grew in the presence of 5 g/l of RLs. Metabolomics and proteomics of the adapted strain grown on CH_4_ in the absence of RLs revealed metabolic changes, increase in fatty acid production and secretion, alterations in gluconeogenesis, and increased secretion of lactate and osmolyte products compared with the parent strain. Expression of plasmid-borne RL production genes in the parent *M. alcaliphilum* strain resulted in cessation of growth and cell death. In contrast, the adapted strain transformed with the RL production genes showed no growth inhibition and produced up to 1 μM of RLs, a 600-fold increase compared with the parent strain, solely from CH_4_. This work has promise for developing technologies to produce fatty acid-derived bioproducts, including biosurfactants, from CH_4_.

## Introduction

Production of surfactants and detergents is a $41.3 billion dollar global industry (https://www.grandviewresearch.com/industry-analysis/surfactants-market). Dominating this field are petroleum-derived chemicals with surfactant properties. Biosurfactants are an attractive class of biomolecules that are sustainable replacements for petroleum-derived surfactants (Chong & Li, 2017; Muller et al., 2012; Sekhon Randhawa & Rahman, 2014). In this group, rhamnolipids (RLs) have been classified as the next-generation biosurfactants (Muller et al., 2012) because they are sustainably produced from renewable resources, are biodegradable, exhibit low toxicity, and are highly reactive as emulsifiers (Muller et al., 2012; Soberón Chávez, 2011). RLs find application in oil recovery and remediation, as antimicrobial and/or antifungal agents, in detergents, cleaners, and agriculture and cosmetics industries (Chong & Li, 2017; Thakur et al., 2021). RLs belong to the class of microbial glycolipids and are predominantly produced at high titer by the opportunistic pathogen *Pseudomonas aeruginosa* (Hauser & Karnovsky, 1957; Jarvis & Johnson, 1949). Therefore, RL biosynthesis, regulation, and bioprocess development have been extensively studied in *P. aeruginosa* (Burger et al., 1963; Chong & Li, 2017; Hauser & Karnovsky, 1957; Lequette & Greenberg, 2005; Thakur et al., 2021).

**Fig. 1:**
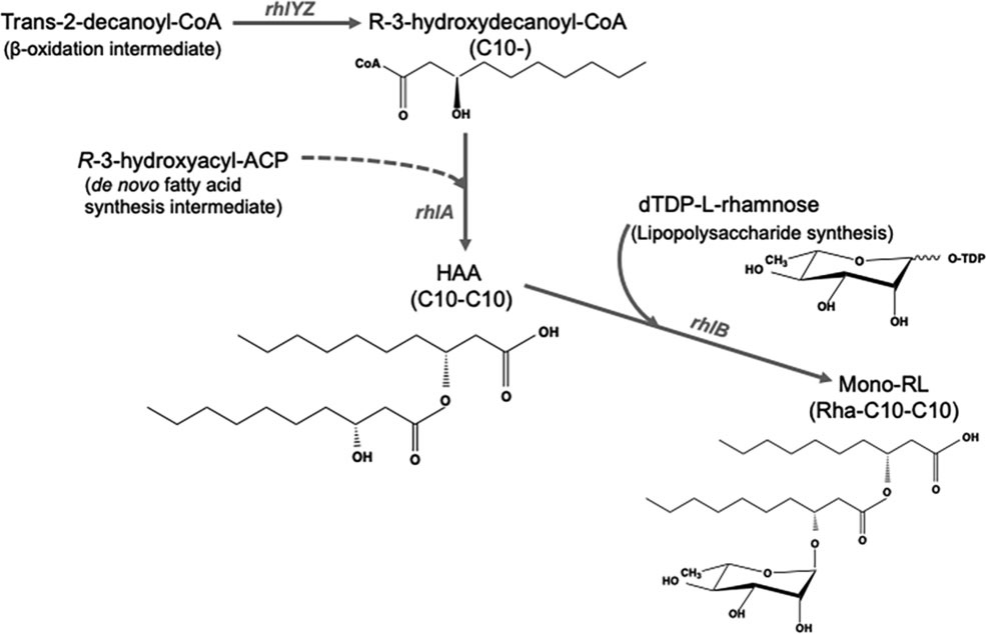
Schematic of rhamnolipid biosynthesis pathway in *Pseudomonas aeruginosa.* HAA, hydroxyakanoyloxyalkanoic acid; *rhlA*, 3-hydroxyacyl-ACP-*O*-3 hydroxyacyltransferase; *rhlB*, rhamnosyl transferase; *rhlYZ*, enoyl-CoA hydratase/isomerase.

RLs are synthesized by diverting intermediates of bacterial fatty acid synthesis or β-oxidation to lipids and subsequently attaching l-rhamnose to the lipid chain, synthesizing the glycolipid (Abdel-Mawgoud et al., 2014). The trans-2-alkanoyl-CoA, an intermediate of the β-oxidation/fatty acid synthesis pathway, is first hydrated and isomerized to *R*-3-hydroxyalkanoyl-CoA by *R*-specific enoyl-CoA hydratase/isomerase (*rhlY, rhlZ*) (Fig. 1). *R*-3-Hydroxalkanoyl-CoA is the direct lipid precursor to β-d (β-d-hydroxyalkanoyloxy)alkanoic acid (HAA), synthesized by the product of *rhlA*, 3-hydroxyacyl-ACP-*O*-3 hydroxyacyltransferase. Following that, rhamnosyl transferase encoded by *rhlB* attaches a rhamnose unit to the HAA chain and subsequently another rhamnose unit can be attached by *rhlC* (rhamnosyl transferase-2), making mono- and di-RL (C_10_–C_10_ HAA dominant in *P. aeruginosa*), respectively (Abdel-Mawgoud et al., 2014). l-Rhamnose is a deoxy sugar and an important component of lipopolysaccharide (LPS) synthesis, so the rhamnose biosynthetic pathway is highly conserved and ubiquitous in both gram-negative and gram-positive bacteria (Giraud & Naismith, 2000). In *P. aeruginosa*, RLs are secreted in the medium to promote quorum sensing, biofilm formation, uptake of less soluble substrates, and to act as virulence factors for the host (Abdel-Mawgoud et al., 2010; Bazire & Dufour, 2014). With medium component and carbon source optimization, titers of >100 g/l of RL have been achieved in *P. aeruginosa* strains (Lovaglio et al., 2010; Trummler et al., 2003; Wei et al., 2005). The high cost of substrates (glucose and additional hydrocarbons) and biosafety concerns related to the pathogenicity of *P. aeruginosa* have limited commercialization in food, agriculture, cosmetic, and pharmaceutical applications (Chong & Li, 2017; Irorere et al., 2017; Muller et al., 2012; Soberón Chávez 2011). Therefore, heterologous expression of RL synthetic pathway or isolation of native RL-producing strains by generally recognized as safe hosts has garnered much interest. *Burkholderia* spp. have been identified as alternative native RL producers (Costa et al., 2011; Dubeau et al., 2009; Hörmann et al., 2010) and *Escherichia coli* and *Pseudomonas putida* have been explored as heterologous hosts that express the *P. aeruginosa* RL biosynthetic genes. A highest RL titer of 7.3 g/l was reported for engineered *P. putida* strains expressing *rhlAB* of *P. aeruginosa* (Beuker et al., 2016; Wittgens et al., 2011). The costs associated with mixed carbon substrates and nitrogen source used in high-titer RL production have been estimated to be 50% of the total production cost (Chong & Li, 2017; Lotfabad et al., 2016) Therefore, lower cost substrates are needed to improve the economics of RL production.

Methane is an abundantly available and low-cost feedstock. It is a component of a fossil source, natural gas, as well as a renewable source, biogas. Considering that methane is a highly potent greenhouse gas (GHG) (Fletcher & Schaefer, 2019; Wuebbles; & Hayhoe, 2002) and one of the main targets for climate-change mitigation, novel technologies for methane utilization are becoming a must element for all industries that produce methane as a by-product. Biogas, a mixture of CH_4_ and CO_2_, is the product of anaerobic digestion, whereas natural gas, found in abundance in the subsurface, is comprised of >90% methane with impurities of volatile higher alkanes (Rahman et al., 2018; Wuebbles & Hayhoe, 2002). Since the United States has substantial reservoirs of natural gas and an increasing capability to produce biogas (Jin et al., 2012; Shen et al., 2015), there is recent interest in methane as a feedstock for microbial conversion (Rahman et al., 2018). Methanotrophs are bacteria that can use methane as a sole carbon source for growth (Chistoserdova et al., 2009; Kalyuzhnaya & Xing, 2018). Recently, some methanotrophs, in particular, *Methylococcus capsulatus* and *Methylotuvimicrobium buryatense*, have emerged as microbial platforms for methane conversion to bio-based chemicals (Clomburg et al., 2017; Henard et al., 2016, 2017). *Methylotuvimicrobium alcaliphilum* (syn. *Methylomicrobium alcaliphilum*) is an attractive methanotrophic host. *Methylotuvimicrobium alcaliphilum* is a gram-negative, haloalkaliphilic, obligate methanotroph with a known genome sequence and for which basic genetic tools for engineering and gene expression have been developed (Akberdin et al., 2018; Henard et al., 2019; Rozova et al., 2010).

In this study, *M. alcaliphilum* was engineered to produce rhamnolipids from CH_4_ without additional mixed or expensive substrate supplementation. The wild type *M. alcaliphilum* strain exhibited inhibited growth when the *P. aeruginosa rhl* genes were expressed; however, adaptation of *M. alcaliphilum* to grow in the presence of RLs produced an evolved strain tolerant to RLs and was able to produce up to 1 µM mono-RL from methane.

## Materials and Methods

### Bacterial Strains, Plasmids, and Growth Conditions

The *E. coli* and *M. alcaliphilum* strains and plasmids used in this study are listed in Table 2. Luria–Bertani (LB) broth and agar plates were routinely used to culture *E. coli* at 37°C. For routine cultivation of *M. alcaliphilum* strain WT (wild type) and its derivatives, Pi (π) media with 3% (wt/vol) NaCl was used as described (Collins & Kalyuzhnaya, 2018) When needed, kanamycin (Kan) was added to the growth medium at 100 µg/ml for *M. alcaliphilum* and 50 µg/ml for *E. coli* cultures. Ampicillin was added to the growth medium at 100 µg/ml. *Methylotuvimicrobium alcaliphilum* cell cultures were grown as batch cultures, either 4 ml culture in 20 ml anaerobic glass tubes or 10 ml culture in 50 ml serum vials, under a methane (99.9%; Airgas):air atmosphere (1:1). Cell cultures were incubated at 30°C, shaking at 220 rpm. Cell growth was measured as optical density (OD 600 nm) using a Spectronic 200E spectrophotometer at the time points mentioned in the Results and Discussion section. Single-colony isolates and transformant selections were performed on Pi media agar plates incubated in anaerobic jars (Oxoid, Remel) under a methane–air atmosphere (1:1). For induction of introduced genes in *M. alcaliphilum* (pDΑ17), the antimicrobial activity of the P_tet_ inducer anhydrotetracycline (aTC) was first evaluated (Fig. S1) with methanol as the substrate. It was observed that aTC’s antimicrobial effect on *M. alcaliphilum* was apparent at concentrations >2.5 µg/ml. Thus, based on genes encoding the RL biosynthetic pathway and a previous report (Henard et al., 2016), a concentration of 1 µg/ml aTC was used for optimal gene expression. *Methylotuvimicrobium alcaliphilum* (pDA17) cultures were induced with aTC (1 µg/ml) added at the time of inoculation (Henard et al., 2016).

### Plasmid Construction and Transformation

The RL biosynthetic genes from *P. aeruginosa* (GenBank RefSeq: NC_002 516.2) containing the genes *rhlY, Z, A*, and *B* encoding *R*-specific enoyl-CoA hydratase/isomerase, 3-hydroxyacyl-ACP-O-3 hydroxyacyltransferase, and rhamnosyl transferase, respectively, were codon optimized for optimal protein production in *M. alcaliphilum* and synthesized by Genscript (Table S1). The codon-optimized *rhl* genes for *M. alcaliphilum* were assembled in concatenation in a replicative expression plasmid, pET28b (+) with individual ribosomal binding sites upstream of each gene. The steps of assembly are illustrated in Fig. S2. The assembled *rhl* cassette was then transferred to the methanotrophic replicative shuttle vector pCAH01. Vector pCAH01 has a P_tet_-driven and aTC-inducible expression system (Henard et al., 2016). For constitutive expression of *rhlYZAB*, the sucrose-phosphate synthase promoter region from the *M. alcaliphilum* WT genome was added upstream of the *rhlYZAB* cassette, replacing the P_tet_ sequence in pCAH01. The final vector constructs pDA17(P_tet_-*rhlYZAB*) and pAD21(P_sps_-*rhlYZAB*) were assembled using Gibson assembly (New England Biolabs). DNA fragments were PCR amplified using Q5 high-fidelity DNA polymerase (New England Biolabs). PCR products were either gel purified or column purified using Qiagen agarose gel or PCR product clean-up kits, respectively. All the PCR primers used for DNA amplification and plasmid construction are listed in Table S2. Assembled plasmids were transformed to *E. coli* Top10 using a chemical DNA transformation method, for propagation, and screened by colony PCR and sequence validated by GENEWIZ sequencing services. Subsequently, plasmids were transformed to *E. coli* strain S17-1 and transferred to *M. alcaliphilum* via conjugation as described previously (Puri et al., 2015).

All the strains and plasmids developed in this work, along with their associated information, have been deposited in the public instance of the Joint BioEnergy Institute (JBEI) Registry (Ham et al., 2012) (https://public-registry.jbei.org/folders/713).

### Adaptive Laboratory Evolution and Development of *M. alcaliphilum* Strain DASS

*Methylotuvimicrobium alcaliphilum* strain DSM19304 was grown in batch cultures of 10 ml Pi media in 50 ml serum vials under a methane–air atmosphere at 30°C with agitation at 200 rpm. To adapt the cells to grow in the presence of RLs, 0.5 g/l RL (90% mono-RL, Millipore Sigma) was supplemented to the starting cell culture medium. The concentration of RLs was increased gradually and stepwise (1, 1.5, 2, 3, 4, and 5 g/l) to achieve a final strain of *M. alcaliphilum* tolerant to 5 g/l RL. A 0.1% inoculum was manually transferred from a growing batch culture to a fresh culture in 48–60 hr. The RL concentration in the media was increased to the next higher concentration when the OD 600 nm at 48 hr of the growing batch culture with RL reached a similar OD_600_ to the WT (>1.0) at the end of 48 hr. After the adaptation, single colonies of *M. alcaliphilum* strain DASS were isolated on Pi media agar plates. Multiple single-colony isolates were confirmed to be *M. alcaliphilum* via 16S rRNA sequencing to rule out co-contaminants. No differences were observed in growth of multiple single colonies that were tested; one clone was selected for further analysis and plasmid transformation.

### Proteomic Analysis

*Methylotuvimicrobium alcaliphilum* cell cultures were grown in batch in 10 ml Pi medium in 50 ml serum vials under a methane–air atmosphere. Cell cultures were incubated at 30°C, shaking at 220 rpm. Both strains were grown for proteomic analysis in triplicates. Cells were harvested at 24 and 48 hr and stored at −80°C until use. Samples for proteomic analysis were processed and whole proteome was analyzed as previously described (dx.doi.org/10.17 504/protocols.io.bf9xjr7n). Normalized spectral abundance factor (NSAF) values obtained were processed to categorize upregulated and downregulated proteins of *M. alcaliphilum* strain DASS and WT. *P*-values < 0.05 for FC > 0.32 were considered significant and are presented in a heat map table or as specified.

### Metabolite Analysis

Growing *M. alcaliphilum* cell cultures (4 ml in Pi medium) in 20 ml anaerobic glass tubes were harvested at 24 and 48 hr of growth. *Methylotuvimicrobium alcaliphilum* strains harboring plasmids were grown with antibiotic and inducer (aTC), as necessary, in the culture medium. Two milliliters of cell culture was centrifuged at 10 000 rpm for 1 min at room temperature (RT). Thereafter, 1 ml supernatant was stored in a separate tube and the rest was discarded. Cell pellets were immediately quenched by adding 250 µl of 4°C cold 100% methanol. Both the supernatant and pellets were stored at −20°C until further processing. All strains, parents and harboring plasmids, were grown in technical triplicates for analysis. To analyze central carbon metabolism and associated metabolites, the cells and supernatants were processed separately using an aqueous methanol extraction method, as described earlier (Baidoo et al., 2019).

Intracellular metabolites were analyzed via liquid chromatography–mass spectrometry [LC–MS; Agilent Technologies 1290 Infinity II ultra-high performance liquid chromatography (UHPLC) system and Agilent Technologies 6545 quadrupole time-of-flight mass spectrometer (MS)] on a ZIC-pHILIC column (150 mm length, 4.6 mm internal diameter, and 5 μm particle size). The UHPLC method used was as described (Baidoo et al., 2019; Kim et al., 2021). For RL analysis, the cell pellets and supernatants were processed using an acidic (HCl) methanol/chloroform precipitation method described previously (Çakmak et al., 2017).

### Analysis of Fatty Acids

Cell cultures were grown in 4 ml Pi media in 20 ml anaerobic glass tubes, under methane–air at 30°C and shaking at 220 rpm. At 24 and 48 hr, 2 ml culture was aspirated and cell pellets were harvested by centrifugation at 8000 rpm for 10 min at room temperature. Supernatants and pellets were stored in separate 2 ml Eppendorf tubes at −80°C until further processing. Total cell fatty acids were analyzed as fatty acid methyl esters (FAMEs) using gas chromatography–mass spectrometry (GC–MS). FAMEs were prepared by transesterification using 2% (vol/vol) sulfuric acid in methanol (90°C; 2 hr). FAMEs were subsequently extracted in 400 μl hexane, of which 1 μl was analyzed on an Agilent 5973 HP6890 GC–MS using a 30 m DB-5 ms capillary column. Electron ionization (EI) GC-MS analyses were performed with a model 7890A GC quadrupole mass spectrometer (Agilent) with a DB-5 fused silica capillary column as described previously (Changhao et al., 2013).

### RL Analysis

The analytes were separated on a Phenomenex Kinetex XB-C18, 3 × 100 mm, 2.6 µm column via an Agilent Technologies high-performance liquid chromatography (HPLC) 1260 system. The sample tray and column compartment were set to 4 and 50°C, respectively. A sample injection volume of 5 μl was used throughout. Mobile phase A was composed of 0.1 % formic acid (Sigma-Aldrich, St. Louis, MO, USA) and 5 μM medronic acid (from the Agilent Technologies InfinityLab Deactivator Additive solution) in LC–MS grade water (Honeywell Burdick & Jackson, Charlotte, NC, USA) and mobile phase B was composed of 0.1% formic acid and 5 μM medronic acid in LC–MS grade methanol (Honeywell Burdick & Jackson, Charlotte, NC, USA). The following gradient was used to separate the analytes: 60 %B at 0 min, linearly increased to 97.1 %B in 4 min, held at 97.1%B for 3 min, linearly decreased to 60 %B in 0.2 min, and held at 60 %B for 3 min. The flow rate was set to 0.42 ml/min from 0 to 7 min, then increased to 0.65 ml/min in 0.2 min, and held at 0.65 ml/min for 3 min. The total HPLC run time was 10.2 min.

The HPLC system was coupled to an Agilent Technologies 6520 quadrupole-time-of-flight (Q-TOF) mass spectrometer (MS). The Q-TOF-MS system was operated via electrospray ionization (ESI) in the negative ion mode at a mass range of 100–1100 *m*/*z* and an acquisition rate of 0.86 spectra/s. The ESI source parameters were set as follows: gas temperature = 330°C, drying gas = 11 l/min, nebulizer = 30 lb/in^2^, VCap = 3500 V, fragmentor = 140 V, skimmer = 50 V, and OCT 1RF Vpp = 170 V. Data acquisition was performed by the Agilent Technologies MassHunter Workstation software and data analysis by the Agilent Technologies MassHunter Qualitative Analysis and Profinder software.

## Results and Discussion

### Impact of RLs on Growth of *M. alcaliphilum*

**Fig. 2:**
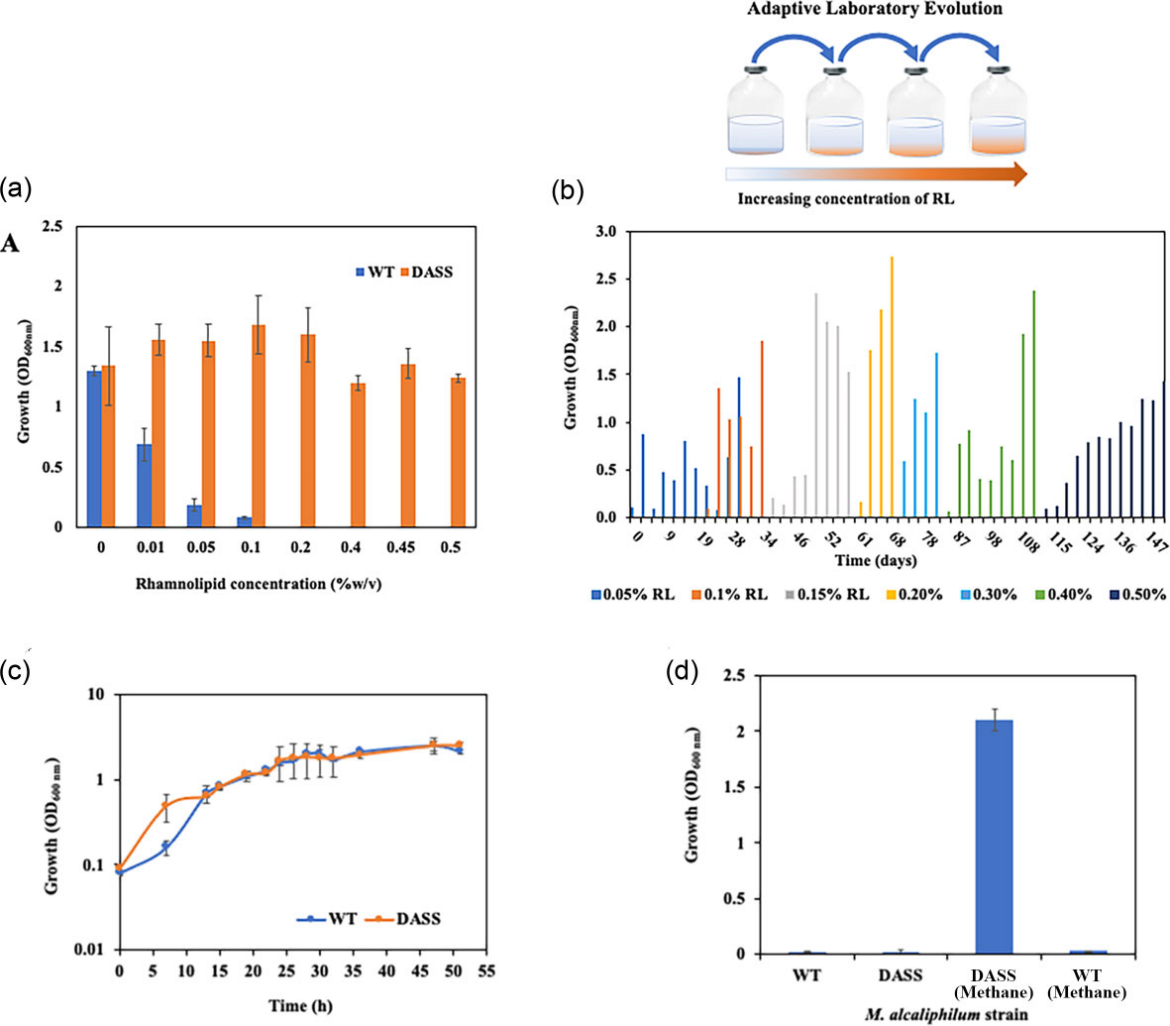
(a) Inhibitory effect of increasing rhamnolipid (RL) concentration on growth of *Methylotuvimicrobium alcaliphilum* strain DSM19304 [wild type (WT)] and strain DASS, grown on Pi medium with CH_4_. (b) Adaptive laboratory evolution of *M. alcaliphilum* by serial transfers in RL containing Pi medium for tolerance. (c) Growth profile of strain WT and DASS in Pi medium with CH_4_. (d) Evaluation of C-source responsible for growth of strains WT and DASS when grown with or without CH_4_ in Pi medium supplemented with 0.5% (wt/vol) RL. WT, wild type strain DSM19304; DASS, RL-tolerant strain created during this work; see text for details.

*Methylotuvimicrobium alcaliphilum* converts methane by sequential oxidation to formaldehyde, which enters the central carbon metabolism through the RUMP pathway (Ojala et al., 2011). *Methylotuvimicrobium alcaliphilum* produces high amounts of glycogen, sucrose, and ectoine with smaller amounts of lactate, formate, succinate, and no known reports of RLs (Akberdin et al., 2018; Kalyuzhnaya et al., 2013, 2015). Rhamnolipids are used as biocontrol/antimicrobial agents, and increasing RL concentrations were found to negatively impact growth of gram-negative and gram-positive heterologous hosts, *E. coli, Bacillus subtilis*, and *Corynebacterium glutamicum* (Wittgens et al., 2011). Therefore, *M. alcaliphilum* growth was tested in the presence of RLs. Compared with the maximum optical density of *M. alcaliphilum* after 36 hr of culture, a 50% reduction in final optical density was observed when the medium was amended with 0.1 g/l RL and almost complete inhibition was observed with 1 g/l RL amendment (Fig. 2a). The RL toxicity to *M. alcaliphilum* is much higher than reported concentrations in other gram-negative hosts like *E. coli* (>90 g/l). The toxicity of RLs to *M. alcaliphilum* required adaptive evolution to permit the strain to produce RLs.

### Adaptive Laboratory Evolution of *M. alcaliphilum*

A course of adaptive laboratory evolution to allow *M. alcaliphilum* to grow on CH_4_ in the RLs was followed for 4 months. During this adaptation, *M. alcaliphilum* strain DSM19304 (hereafter referred to as “WT,” wild type) was subjected to gradually increasing RL concentrations starting from 0.5 to 5 g/l (Fig. 2b). At the end of multiple transfers over a period of 4 months, an *M. alcaliphilum* strain tolerant to RLs (strain DASS) was obtained. *Methylotuvimicrobium alcaliphilum* strain DASS tolerated 5 g/l RLs with comparable final optical density (OD_600nm_) and growth profile to the WT strain (Fig. 2a and c). Incubation of the DASS strain in 5 g/l RL did not promote growth in the absence of CH_4_, indicating that *M. alcaliphilum* did not adapt to grow with RLs as a carbon source (Fig. 2d).

### Strain Characterization

To discern the phenotypic difference between the WT and DASS strains, GC-MS analyses of fatty acids, proteomics, and targeted metabolomics were performed on both strains grown on CH_4_ in the absence of RLs. The results of these experiments are presented and discussed.

#### Fatty acid assessment

Fatty acids are a vital component of microbial cells, which are used as building blocks to construct cell membranes, as well as to provide precursors for synthesis of storage, energy, and signaling molecules (de Carvalho & Caramujo, 2018). Surfactants and detergents solubilize the lipids of the membrane and disrupt the cell structure (Jones, 1999). Therefore, *M. alcaliphilum* DASS may have alterations in its fatty acid and/or lipid biosynthesis that enabled the strain to tolerate higher RL concentrations relative to the WT strain. The approach was to establish preliminary evidence for this possibility by quantifying long-chain (LC) fatty acids produced by the strains grown on CH_4_. LC fatty acids (>C_12_) are known precursors to phospholipids (PLs) and LPSs that constitute the cell membrane (de Carvalho & Caramujo, 2018). Moreover, type-I methanotrophs, including *M. alcaliphilum*, are known to contain mainly 16:0 and 16:1 fatty acids (Bowman et al., 1991; Costello et al., 2002). GC-MS analysis was performed on the 24 and 48 hr cultures focusing on C_16_ and C_18_ fatty acids that are involved in PL and LPS synthesis (Table 1).

**Table 1.** Fatty Acid Methyl Ester (FAME) Content of *Methylotuvimicrobium alcaliphilum* Strains DSM19304 [Wild Type (WT)] and DASS

	Strain DASS				Strain WT			
	Supernatant (nM)		Intracellular (nM)		Supernatant (nM)		Intracellular (nM)	
Fatty acid	24 hr	48 hr	24 hr	48 hr	24 hr	48 hr	24 hr	48 hr
C16:0	UD	0.14 ± 0.01	28.71 ± 8.15	101.93 ± 11.46	UD	UD	23.68 ± 2.42	49.72 ± 20.47
C16:1	19.04 ± 5.88	15.89 ± 0.92	12.57 ± 2.50	14.54 ± 1.56	2.92 ± 0.45	3.19 ± 0.63	8.09 ± 1.16	9.18 ± 0.78
C18:1	19.07 ± 7.15	13.89 ± 1.13	7.92 ± 4.25	8.22 ± 1.86	UD	UD	2.63 ± 0.38	2.28 ± 1.74
C18:2	26.14 ± 6.58	12.96 ± 0.43	15.19 ± 4.66	15.72 ± 4.78	13.27 ± 1.53	14.76 ± 2.71	17.32 ± 7.44	23.75 ± 8.76
C20:0	23.70 ± 2.68	14.77 ± 2.02	15.98 ± 3.45	14.46 ± 2.82	7.16 ± 0.59	7.99 ± 1.60	31.23 ± 4.16	25.45 ± 5.78

*Note.* Cells were cultivated in 4 ml Pi media, in 20 ml anaerobic glass tubes at 30°C and shaking at 220 rpm under methane:air (1:1) vol/vol. UD, undetectable, below 0.5–0.8 nM depending on the FAME.

A relatively high abundance of C_16:0_ fatty acid was observed in the cell pellets of both strains, WT and DASS (Table 1), which is consistent with previous findings of other type-I methanotrophs (Bowman et al., 1991). However, when strains WT and DASS are compared with each other, C_16:0_ concentrations were ∼2× higher in strain DASS in the cell pellet at 48 hr. The C_16:1_ fatty acid concentration was found to be 1.5× higher in cell pellets and 5–6× higher in the supernatant of strain DASS compared with the WT (Table 1). Also, C_18:1_ was undetected in the supernatant of the WT strain but found at a similar abundance to that of the C_16:1_ fatty acid in the DASS strain. Therefore, the DASS strain produces higher amounts of fatty acids than the WT strain and secretes them at higher levels into the medium. Excretion of free fatty acids is not a regular occurrence in methanotrophic bacteria (Kalyuzhnaya & Xing, 2018). It is proposed, in strain DASS, that to maintain cell membrane integrity from solubilizing in the surfactant, a high rate of fatty acid synthesis be maintained to continually replenish PL and LPS layers of the cell membrane, as suggested by the observed high C_16:0_, C_16:1_, and C_18:1_ fatty acids in the cell pellet (Table 1). At the same time, to maintain a normal lipid to protein ratio for cell homeostasis, excess fatty acids must be secreted out or stored as intracellular granules [like polyhydroxyalkanoates (PHAs)] (Parsons & Rock, 2013). Since type-1 methanotrophs are known to accumulate glycogen and not PHAs, the outlet for the excess fatty acids in this host is excretion. The possibility of enhancing the secretion of free fatty acids has been explored by engineering many microbial platforms (Lennen & Pfleger, 2013). *Methylotuvimicrobium alcaliphilum* DASS is innately capable of improved fatty acid production and could serve as a foundational strain for further development of a fatty acid-based biofuel/chemical production platform from CH_4_.

#### Metabolite and proteome analysis of M. alcaliphilum strains DASS and WT

**Fig. 3:**
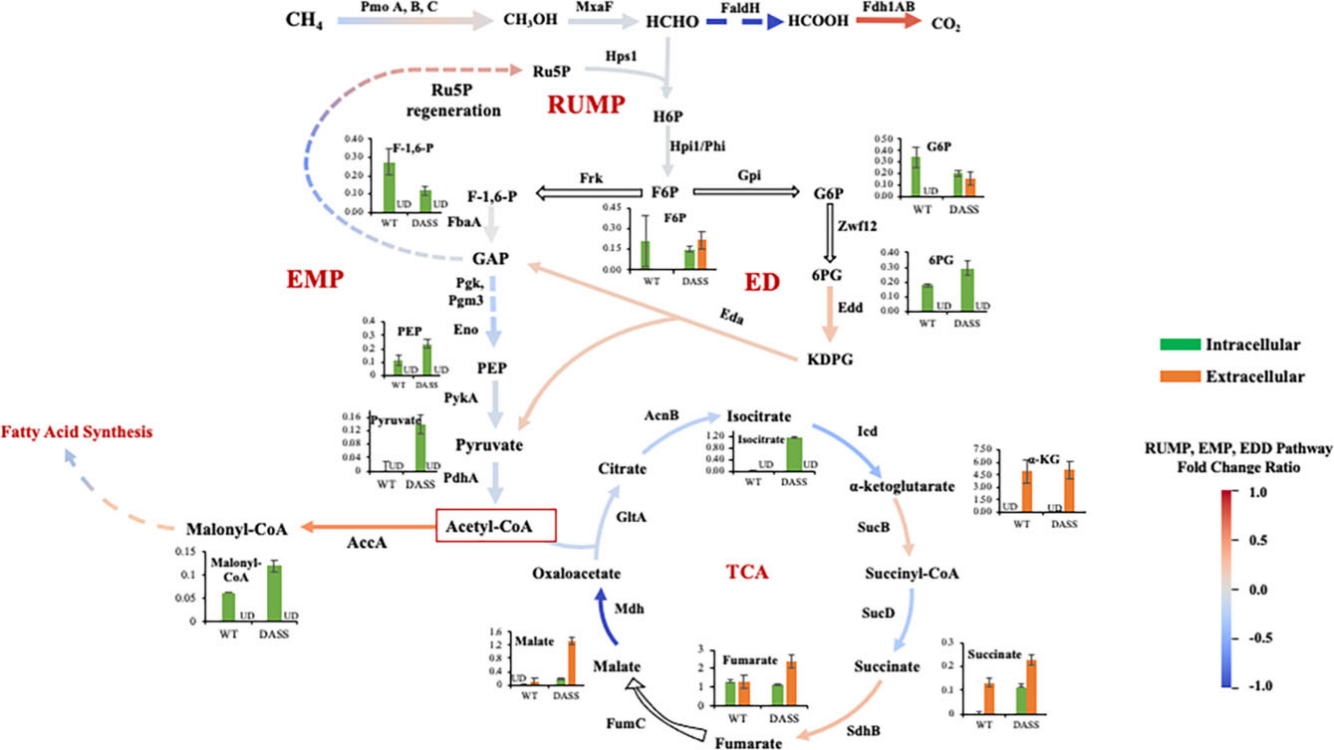
Schematic of differential expression of proteins and metabolites of ribulose monophosphate (RuMP), Embden–Meyerhof–Parnas (EMP), and Entner–Doudoroff (ED) pathway in *Methylotuvimicrobium alcaliphilum* wild-type (WT) and DASS strains at 24 hr of growth on methane. Pathway arrows represent the fold change ratio of average normalized spectral abundance factor (NSAF) values of two independent experiments of DASS over WT strain [(NSAF_DASS_ − NSAF_WT_)/NSAF_WT_]. The fold change ratio is the ratio of change in final (NSAF_DASS_) and original (NSAF_WT_) value over original value, where a fold change ratio of 1 would mean a change by two times of the original value, and a fold change ratio of −0.5 will correspond to the final value being half of the original value. Graphs depict the absolute concentration of metabolite quantified in µM (*Y*-axis) from three independent experiments. AccA, acetyl-CoA carboxylase; AcnB, aconitate hydratase; Eda, aldolase; Edd, dehydratase; Eno, enolase; FaldH, formaldehyde dehydrogenase; FbaA, fructose-bisphosphate aldolase, class II; Fdh1A&1B, NAD-dependent formate dehydrogenase, alpha and beta subunit; FumC, fumarate dehydrogenase; GltA, citrate synthase; Gpi, phosphoglucose isomerase; Hps1, 3-hexulose-6-phosphate synthase; Hpi1/Phi, 3-hexulose-6-phosphate isomerase; Icd, isocitrate dehydrogenase; MtkB, succinate-CoA synthetase; MxaF, methanol dehydrogenase; PdhA, pyruvate dehydrogenase E1 component; Pgk, phosphoglycerate kinase; Pgm3, phosphoglycerate mutase; PmoA, B &C, particulate methane monooxygenase, subunit A, B, and C; PykA, pyruvate kinase; Mdh, malate dehydrogenase; Sdh, succinate dehydrogenase; SucB, α-ketoglutarate dehydrogenase; Zwf, glucose dehydrogenase; F-1,6-P, fructose-1,6-bisphosphate; F6P, fructose-6-phospahte; GAP, glyceraldehyde-3-phosphate; G6P, glucose-6-phosphate; H6P, hexulose-6-phosphate; KDPG, 2-dehydro-3-deoxyphosphogluconate aldolase; PEP, phosphoenolpyruvate; 6PG, 6-phosphogluconate; Ru5P, ribulose-5-phosphate. Green, intracellular concentration (µM); orange, extracellular concentration (µM); UD, undetectable below 2–20 nM depending on the metabolite.

To study the physiological variations that have occurred due to the surfactant tolerance of the newly adapted strain DASS with respect to its parent, the metabolome and proteome of strain DASS were analyzed and compared with those of strain WT. The concentration of select metabolites in intracellular and extracellular fractions of 24 hr old cultures was determined using LC-MS. Presented in Fig. 3 is a schematic of central carbon metabolism with arrows depicting the fold change ratio of selected peptides as a heat map and absolute metabolite concentrations (µM) in graphs. Though *M. alcaliphilum* harbors genes of the Entner–Doudoroff (ED) and Embden–Meyerhof–Parnas (EMP) pathways, it has been reported previously that methane metabolism is through the ribulose monophosphate (RuMP)-EMP route (Kalyuzhnaya et al., 2013) (Fig. 3). However, the overlying metabolite concentrations and proteome indicate that in strain DASS, the ED route is preferred over and in adjunction to EMP, for growth. A comparatively lower concentration of fructose-1,6-phosphate (EMP intermediate) and higher concentration of 6-phosphogluconate (ED intermediate) in strain DASS versus WT was observed, supported by a modest increase in abundance of proteins (20%) encoding for the key enzymes of the ED pathway, 6-phosphogluconate dehydratase (Edd) and 2-dehydro-3-deoxy-phosphogluconate aldolase (Eda). It is proposed that 6PG was subsequently converted to 2-dehydro-3-deoxy-phosphogluconate (KDPG), followed by conversion to pyruvate and glyceraldehyde-3-phosphate (GAP) via Edd and Eda, respectively. Considering that KDPG accumulation might inhibit cell growth, it was apparently converted to pyruvate (1.5-fold in DASS vs. WT) and GAP (Fuhrman et al., 1998). Though GAP was not detected, the higher concentration of the phosphoenolpyruvate (PEP) pool in strain DASS is suggestive of higher GAP levels. Also, phosphorylated sugar intermediates were detected, like fructose-6-phosphate (F6P) and glucose-6-phosphate (G6P) in the culture medium of strain DASS only, which might be a stress response and warrants further investigation (George et al., 2018; Zhou et al., 2012). Among intermediates of the TCA cycle, higher amounts of succinate, fumarate, and malate were observed in the cells and in the culture medium compared with the WT (Fig. 3). According to proteomic data, malate dehydrogenase (Mdh), which catalyzes the conversion of malate to oxaloacetate, was downregulated in strain DASS. The reduction of carbon flux via Mdh could explain the observed 1.2-fold higher levels of secreted malate in the newly evolved strain (Fig. 3). Moreover, a twofold higher internal malonyl-CoA concentration was detected in strain DASS, a direct precursor to the fatty acid biosynthesis pathway. An increase of 50% in AccA protein abundance further supports the finding of increased fatty acid biosynthesis by strain DASS (Fig. 3 and Table 1).

**Fig. 4:**
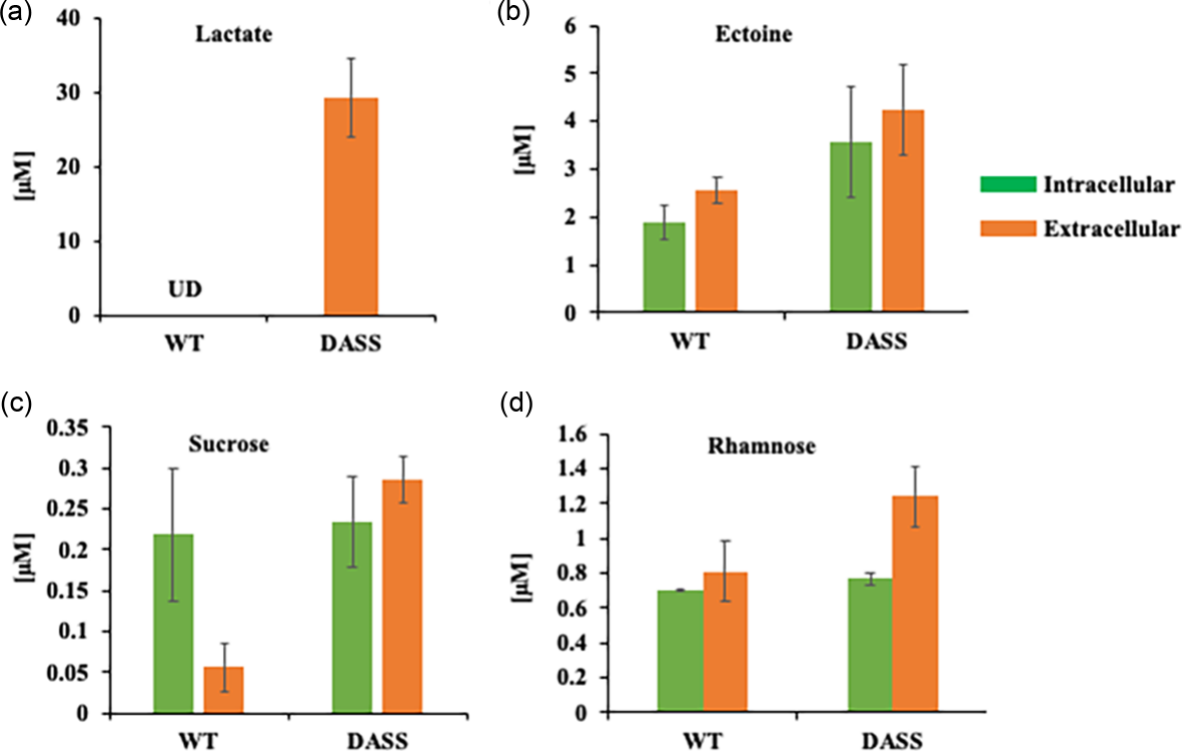
Absolute metabolite concentrations detected in strains wild type and DASS. (a) Lactate, (b) ectoine, (c) sucrose, and (d) rhamnose. Cells were cultivated in 4 ml Pi media, in 20 ml anaerobic glass tubes at 30°C and shaking at 220 rpm, under methane:air (1:1) vol/vol. Green, intracellular concentration; orange, extracellular concentration; UD, undetectable below 2 nM.

Other secreted products included lactate as well as sucrose and ectoine. Another key metabolite, rhamnose, was also evaluated since it is a native precursor of interest for heterologous RL synthesis as well as being involved in LPS biosynthesis. Lactate was undetected in the WT strain but present at ∼30 μM in the extracellular fraction from strain DASS (Fig. 4). Both ectoine and sucrose are well-characterized osmoprotectants synthesized by *M. alcaliphilum* typically in response to high salinity and alkalinity of the medium (But et al., 2015; Kalyuzhnaya et al., 2013; Mustakhimov et al., 2010). In strain DASS, the secreted sucrose level was detected to be ∼30-fold higher, though the internal sucrose concentration was found to be unaffected. Overall ectoine production was found elevated in strain DASS with ∼1.6-fold increase with respect to the WT. Moreover, although the intracellular concentration of rhamnose was unchanged, rhamnose secretion was found to be 1.1-fold higher in strain DASS compared with the WT (Fig. 4).

**Fig. 5:**
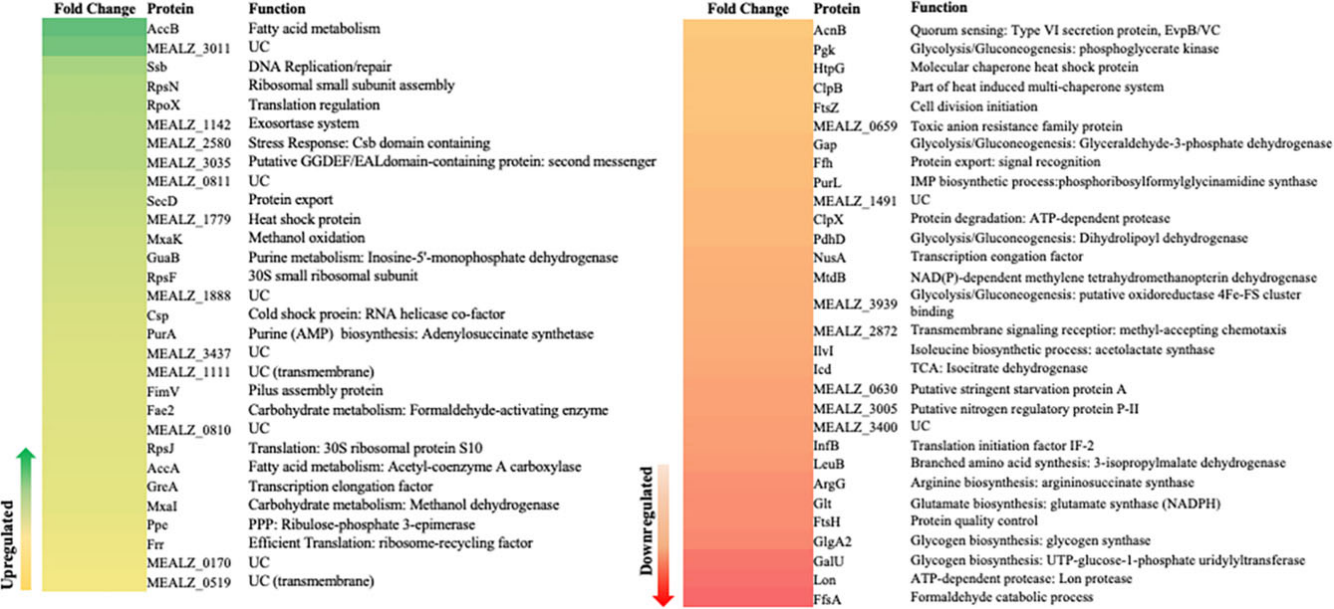
Heat map representing the fold change of peptide count [NSAF_DASS_/NSAF_WT_] (NSAF, normalized spectral abundance factor) in strain DASS compared with wild type at 24 hr of growth. UC, hypothetical and/or uncharacterized proteins; UC (transmembrane), uncharacterized protein with transmembrane signal peptide domain. Yellow to green, significantly upregulated (2.3  ≥ FC ≥ 0.32); orange to red, significantly downregulated (−0.32 ≥ FC ≥ −1.8).

On evaluation of the whole cell proteome of the strains, a total of 725 proteins were detected at the two experimental time points. Out of the total, 118 proteins were observed to be downregulated and 102 were found upregulated; however, after qualifying *p* ≤ 0.05 and log_2_ FC ≥ 0.32 value significance test, only 30 proteins were characterized as significantly down- and upregulated, respectively, at 24 hr. The fold change in NSAF of proteins in strains DASS and WT at 24 hr is listed and represented as a heat map in Fig. 5. As listed in Fig. 5, at 24 hr, a more than 200% increase in acetyl-CoA carboxylase subunit AccB and 50% increase in subunit AccA were observed. These subunits are involved in the synthesis of malonyl Co-A from acetyl-CoA, a direct precursor of the fatty acid synthesis pathway (Demidenko et al., 2016). Also, an increase was detected in abundance of proteins involved in translation, export, and quality control machinery (RpsN, RpsF, RpsJ, RpoX, Frr, MEALZ_1142, and SecD), and many uncharacterized proteins with transmembrane domain and OmpA-like outer membrane domain (MEALZ_1111, 0519) (Fig. 5). Apparently, the adaptation to overcome environmental stress to the surfactant resulted in an increase in abundance of heat shock and other stress response proteins and chaperonins (MEALZ_1779, 2580, Csp) in strain DASS. Additionally, an increased abundance in transcription factors (GreA), DNA replication/repair proteins (Ssb, GuaB, and PurA) and ion-exchange/cell-response regulators (MEALZ_3035) to maintain cellular homeostasis was also identified. Moreover, a >50% increase was observed in the protein abundance for carbohydrate metabolism, methanol and formaldehyde oxidation (MxaK, Mxal, and Fae2), and ribulose monophosphate pathway enzyme expression for methane metabolism (Ppe). However, enzymes involved in glycolysis/gluconeogenesis (FfsA, MEALZ_2872, Mtb, PdhD, Gap, Pgk) were downregulated (Fig. 5), which is also reflected in the central carbon metabolite data (Fig. 3). Also, lower abundances of proteins were observed in the glycogen biosynthetic pathway enzymes (GlgA2 and GalU), which is in agreement with the diversion of central carbon to ectoine, lactate, and sucrose by strain DASS. A similar observation was supported with results from 48 hr old cultures (Fig. S3).

Based on the metabolomic and proteomic data, at 24 hr of cultivation, the DASS strain shifted central carbon processing from EMP to ED, with the simultaneous activity of both pathways contributing to higher pyruvate pools. The observation of lactic acid secretion by strain DASS is likely resulting from the increased internal pyruvate pool. This work on strain DASS identified the unique metabolic changes due to surfactant acclimatization that reinforced evidence of the increased pool of fatty acids and rhamnose, a positive outcome for engineering this strain for RL biosynthesis.

### RL Biosynthesis

*Methylotuvimicrobium alcaliphilum* is not known to produce RLs, so it was essential to identify the availability of precursors for heterologous RL synthesis in this host. Including the four gene (*rhlYZAB*) enzyme cassette from *P. aeruginosa*, the prerequisites for RL production are fatty acid biosynthesis/β-oxidation and an available pool of rhamnose. Fatty acid biosynthesis is well characterized for *Methylotuvimicrobium buryatense* 5GB(1), a methanotroph closely related to *M. alcaliphilum* (Yu et al., 2018); however, reports of *R-*3-hydroxydecanoyl-CoA (direct precursor to RLs) and enzymes for RL synthesis are not known. Internal rhamnose pools have been reported earlier in *M. alcaliphilum* (Akberdin et al., 2018); additionally, the rhamnose concentration was also established during DASS strain characterization (Fig. 4d).

#### Heterologous RL *production in M. alcaliphilum strain WT (parent)*

**Fig. 6:**
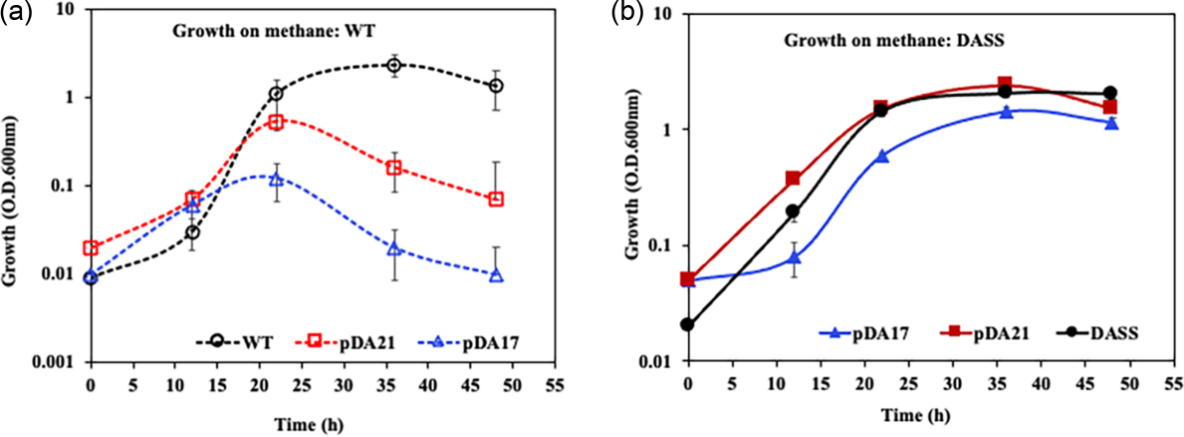
(a) Comparison of growth of *Methylotuvimicrobium alcaliphilum* strains wild type (WT) and WT harboring plasmids pDA17 and pDA21. (b) Comparison of growth of *M. alcaliphilum* strains DASS and DASS harboring plasmids pDA17 and pDA21. Cells were grown as batch cultures in 4 ml Pi media, in 20 ml anaerobic glass tubes at 30°C and shaking at 220 rpm, under methane:air (1:1) vol/vol. Dashed lines and hollow markers, WT; solid lines and markers, strain DASS; black circles, parent strains; blue triangles, pDA17; red squares, pDA21.

Codon-optimized *rhlYZAB* was cloned in shuttle vector pCAH01 under inducible (P_tet_: tetracycline; pDA17) and constitutive (P_sps_: sucrose phosphate synthase; pDA21) promoters (Table 2). The inducible P_tet_ promoter has been shown to express heterologous *ldh* (lactate dehydrogenase) in type-1 methanotrophs for lactic acid production (Henard et al., 2016), and the constitutive *mxaF* (methane monooxygenase, MMO) promoter has been used for heterologous production of 2,3-butanediol (Nguyen et al., 2018). In this work, for pDA21, *P. aeruginosa rhlYZAB* expression was controlled by the constitutive *M. alcaliphilum* sucrose phosphate synthase promoter (P_sps_), since *M. alcaliphilum* accumulates high amounts of sucrose in its environment in response to maintaining osmotic balance (Fig. 4c). The resulting plasmid constructs with *rhlYZAB* under P_tet_ (pDA17) and P_sps_ (pDA21) were introduced in *M. alcaliphilum* via conjugation and the strains were monitored for growth and RL production. *Methylotuvimicrobium alcaliphilum* WT and WT harboring plasmids pDA17 and pDA21 were cultured in methane and monitored for growth, where *M. alcaliphilum* (pDA21) and strain WT were grown without any inducer. Poor growth was observed for *M. alcaliphilum* (pDA17) and (pDA21) cultures compared with strain WT (Fig. 6a), with optical densities of 0.12 ± 0.05, 0.53 ± 0.11, and 1.1 ± 0.31, respectively. A detectable amount of mono-RL was produced; however, the titers were low, with the pDA21 strain producing 63 nM of RL (Table 3). The observation of cell lysis in the WT cultures with plasmids where the RL production genes are expressed is indicative that the gene products and/or the RLs are toxic to *M. alcaliphilum* WT (Fig. 6a).

**Table 2.** Bacterial Strains and Plasmids Used in the Study

Strains and plasmids	Characteristics	Source
Strains:		
*Escherichia coli* TOP10	F^–^ *mcrA* Δ(*mrr-hsdRMS-mcrBC)* φ80*lacZ*Δ*M15*Δ*lacX74 recA1 araD139 (ara-leu)7697 galE15 galK16 rpsL endA1 λ^–^*	Invitrogen
*E. coli* S17-1	Tp^r^ Sm^r^ *recA thi pro hsd*(r^–^ m^+^)RP4-2- Tc::Mu::Km Tn7	JBEI collection
*Methylotuvimicrobium alcaliphilum* 20Z (DSM19304)	Wild type	DSMZ (JPUB_019 705)
*M. alcaliphilum* DASS	Tolerant to rhamnolipid	This work (JPUB_019 708)
Plasmids:		
pCAH01	P*_tetA_ bla-tetR oriR_CoEI_ _oriRRP4/RK2_, oriT_RP4/RK2_, trfA ahp*	Henard et al. (2016)
pET28b (+)	*E. coli* expression vector *kanR*	Novagen
pUC57	*E. coli* cloning vector *ampR*	Genscript
pDA15	pET28b(+) P_T7_ *rhlY rhlZ rhlA rhlB*	This work (JPUB_019 714)
pDA17	pCAH01 P_tet_ *rhlY rhlZ rhlA rhlB*	This work (JPUB_019 715)
pDA21	pCAH01 P_sps_ *rhlY rhlZ rhlA rhlB*	This work (JPUB_019 717)

*Note.* All strains and plasmids constructed in this work and their related information can be found in the JBEI registry (https://public-registry.jbei.org/folders/713).

**Table 3.** Rhamnolipid Titer Obtained by *Methylotuvimicrobium alcaliphilum* Strains wild type and DASS

			RL (nM)	
Strain (plasmid)	Time (hr)	OD_600 nm_	Intracellular	Extracellular
WT (pDA17)^a,b^	24	0.12 ± 0.01	7 ± 0.01	10 ± 0.01
WT (pDA21)^a,c^	24	0.53 ± 0.11	2 ± 0.01	61 ± 0.01
DASS (pDA17)^a^	24	1.33 ± 0.11	119 ± 0.01	315 ± 0.06
	48	1.45 ± 0.30	367 ± 0.03	293 ± 0.05
DASS (pDA21)^b^	24	1.65 ± 0.10	621 ± 0.08	135 ± 0.03
	48	1.55 ± 0.10	871 ± 0.15	132 ± 0.01

*Note.* Cells were cultivated as batch cultures in 4 ml Pi media, in 20 ml anaerobic glass tubes at 30°C and shaking at 220 rpm, under methane:air (1:1) vol/vol. pDA17 cultures were induced with addition of 1 µg/ml anhydrotetracycline. OD (optical density) and RL (rhamnolipid) values at 24 hr. WT, wild type.

^a^48 hr time point for WT (plasmid) culture was not processed due to cell lysis.

^b^P_tet_ promoter.

^c^P_sps_ promoter driving *rhlABYZ* expression.

#### Heterologous RL production in M. alcaliphilum strain DASS

The toxicity observed when the RL production genes were expressed in *M. alcaliphilum* suggested that the DASS strain might be more amenable to RL production. Though expression of the *rhlYZAB* cassette in strain DASS containing pDA17 and pDA21 had negligible impact on the final culture densities, as apparent in Fig. 6b, strain DASS (pDA17) had a 12 hr lag in growth when compared with DASS and DASS (pDA21). Apparently, the expression of the RL pathway under an inducible promoter impacted the growth of DASS (pDA17), as evident from the negligible impact on the growth of RL under constitutive P_sps_ without an inducer (pDA21; Fig 6b) and the empty vector pCAH01 with an added inducer (Fig. S4). In strain DASS, RLs were produced at 100-fold (pDA17) and 600-fold (pDA21) higher titer, respectively, than in strain WT containing the plasmids (Table 3), with strain DASS (pDA21) producing 1 µM of RLs (0.65 mg/l). However, strain DASS (pDA17) reported the highest secreted concentration of RL at ∼300 nM, which was achieved after 24 hr. From 24 to 48 hr, the RLs in the pDA17 strain accumulated intracellularly. The increase in RL titer observed for the DASS strains is consistent with the increased tolerance to RL obtained by adaptive evolution as well as the increased production of free fatty acids that are the precursors for RL production. In the future, metabolic pathway engineering of strain DASS to eliminate coproduct synthesis, like lactic acid, sucrose, or ectoine, and β-oxidation (▵*fadABE*) can be evaluated for their impact on improving the RL titer. Additionally, continuous-flow bioreactor processes can be performed to obtain a high titer of RL and compute rates and yields of RL production from CH_4_, as has been shown for other bioproducts from methanotrophs (Fei et al., 2014; Henard et al. 2016, 2019). Moreover, other heterologous genes and their expression can be assessed under a constitutive P_sps_ promoter for its effectiveness in continued product synthesis in sucrose-producing methanotrophic platforms.

## Conclusion

The work presented here is a proof-of-concept study to produce RLs from CH_4_. This study demonstrated that RLs inhibit the growth of *M. alcaliphilum*; however, after adaptive laboratory evolution of *M. alcaliphilum* on gradually increasing RL concentrations, *M. alcaliphilum* metabolism was able to grow in the presence of 10-fold higher concentrations of RLs compared with the parent strain. It was also established that the metabolic changes directly impacted fatty acid synthesis in the cells and strain DASS was found to have acquired natural ability to secrete ∼5-fold higher fatty acids in the medium than the parent strain. The strategy of adaptive laboratory evolution enabled the newly generated strain DASS to produce an ∼600-fold higher titer of RL compared with strain WT, where the latter failed to survive when expressing the recombinant RL biosynthetic pathway. The increased fatty acid biosynthesis and secretion by strain DASS suggests a route to develop methanotrophic strains with higher levels of fatty acid production from CH_4_. Genome sequencing will establish the causative mutations, which may be applied to developing strains that produce fatty acid-derived fuels and bioproducts.

## Supplementary Material

kuac002_Supplemental_File
