# Supplementary material for: Adaptive evolution of Methylotuvimicrobium alcaliphilum to grow in the presence of rhamnolipids improves fatty acid and rhamnolipid production from CH4
Source: J Ind Microbiol Biotechnol. 2022 Feb 3;49(2):kuac002. doi: 10.1093/jimb/kuac002 (PMC9118986; doi:10.1093/jimb/kuac002)
Supplement: kuac002_Supplemental_File [file kuac002_Supplemental_File.docx]

**Adaptive evolution of *Methylotuvimicrobium alcaliphilum* to grow in the presence of rhamnolipids improves free fatty acid and rhamnolipid production from CH_4_**

Deepika Awasthi^1^, Yung-Hsu Tang^1^, Bashar Amer^1^, Edward E.K. Baidoo^1^, Jennifer Gin^1^, Yan Chen^1^, Christopher Petzold^1^, Marina Kalyuzhanaya^2^ and Steven W. Singer^1*^

^1^Biological Systems and Engineering Division, Lawrence Berkeley National Laboratory, Berkeley, CA, ^2^Department of Biology, San Diego State University, San Diego, CA.

*Corresponding Author

Dr. Steven W. Singer

Biosystems Design and Engineering Division

Lawrence Berkeley National Laboratory

Berkeley, CA 94720 (U.S.A.)

Email: [SWSinger@lbl.gov](mailto:SWSinger@lbl.gov)

**Supporting Information**

**Materials**

All chemicals used in this study were analytical grade. Organic and inorganic chemicals were purchased from Fisher Scientific (Pittsburgh, PA). Biochemicals were from Sigma-Aldrich Co. (St. Louis, MO) and Millipore Sigma (Burlington, MA). Molecular biology reagents and supplies were from New England Biolabs (Ipswich, MA) and Thermo Fisher Scientific (Waltham, MA). Plasmid DNA extraction kit was from QIAGEN (Valencia, CA). DNA clean up kits were from QIAGEN (Valencia, CA). DNA oligonucleotides for PCR were from IDT (Coralville, IA). Gene synthesis was from GenScript, Piscataway, NJ).

**Table S1. List of PCR primers**

| Name | Sequence (5’-3’) | Usage |
| --- | --- | --- |
| DA11-For | cgcacatatgAGAAGAGAATCGCTGCTT | Codon optimized *rhlA* |
| DA11-Rev | ttacggatccgctagcTTACGCGTATCCTATAGCCATCT |  |
| DA12-For | ggcacatatgCACGCGATACTGATAGCCATA | Codon optimized *rhlB* |
| DA12-Rev | ctatggatccgctagcTTACGACGCAGCCTTCAGCCAT |  |
| DA13-For | cgcacatatgAACACAGCCGTGGAACCTTA | Codon optimized *rhlY* |
| DA13-Rev | ctatggatccgctagcTTAGCAGTTTCTCCACTTCGGGTCTC |  |
| DA14-For | ggcacatatgAACGTGCTGTTTGAAGAGA | Codon optimized *rhlZ* |
| DA14-Rev | ctatggatccgctagcTTACAGTCCGGCCAGCGGATGCT |  |
| DA18-For | CGGATATAGTTCCTCCTTTCA | Validating insert at multiple cloning site (pET28b+) |
| DA18-Rev | GGATAACAATTCCCCTCTAGAA |  |
| DA31-For | AAGCTTGACCTGTGAAGTG | pCAH01 backbone for Gibson assembly of pDA17 |
| DA31-Rev | TTCACTTTTCTCTATCACTGATAG |  |
| DA32-For | cctatcagtgatagagaaaagtgaaTGAACACAGCCGTGGAAC | *Rhl* cassette from pDA15 for Gibson assembly of pDA17 |
| DA32-Rev | atttttcacttcacaggtcaagcttCTTACGACGCAGCCTTCAG |  |
| DA33-For | CATGTTCTTTCCTGCGTTATCC | Validating insert at cloning site (pCAH01) |
| DA33-Rev | AGATCCGTGACGCAGTAG |  |
| DA44-For | TGGTGTCGGGTCATGTGAG | pCAH01 without P_tet_-amp for Gibson assembly for pDA21 |
| DA44-Rev | GAAAATTGTCGGGAAGATGC |  |
| DA45-For | ctggccttttgctcacatgacccgacaccaGGTACTCAAAAAGCCGGTC | P_sps_ from *M. alcaliphilum* genome |
| DA45-Rev | ccacggctgtgttcaTCACGAACAACTATCTCAAG |  |
| DA46-For | gatagttgttcgtgaTGAACACAGCCGTGGAAC | *Rhl* cassette from pDA15 for Gibson assembly of pDA21 |
| DA46-Rev | atcagatcacgcatcttcccgacaattttcTTACGACGCAGCCTTCAG |  |

Homology tails for Gibson assembly and restriction enzyme sites are in lower case

**Table S2: Codon-optimized nucleotide sequence**

| Gene | Optimized nucleotide sequence |
| --- | --- |
| *rhlA* | ATGAGAAGAGAATCGCTGCTTGTGAGTGTGTGCAAAGGACTGAGAGTGCACGTGGAGAGAGTGGGACAGGACCCTGGTAGATCGACAGTGATGCTGGTGAACGGTGCTATGGCGACAACGGCTAGTTTTGCGAGAACGTGCAAATGCCTGGCTGAACACTTTAACGTGGTGCTGTTTGATCTGCCTTTTGCGGGACAGTCGAGACAGCATAACCCGCAGAGAGGTCTGATAACAAAGGACGATGAAGTGGAGATACTGCTGGCCCTGATAGAAAGATTTGAGGTGAACCATCTGGTGTCGGCGAGTTGGGGAGGGATAAGTACGCTGCTGGCCCTGAGTAGAAACCCTAGAGGGATAAGATCGAGTGTGGTGATGGCCTTTGCTCCGGGGCTGAACCAGGCTATGCTGGACTACGTGGGAAGAGCGCAGGCCCTGATAGAACTGGACGATAAGAGTGCGATAGGTCACCTGCTGAACGAGACAGTGGGGAAATACCTGCCTCAGAGACTGAAGGCGTCGAACCACCAGCACATGGCCAGTCTGGCTACGGGAGAATACGAGCAGGCCAGATTTCATATAGACCAGGTGCTGGCCCTGAACGATAGAGGTTACCTGGCTTGCCTGGAGAGAATACAGTCGCACGTGCATTTTATAAACGGGAGTTGGGACGAATACACAACGGCGGAGGACGCCAGACAGTTTAGAGATTACCTGCCGCACTGCTCGTTTAGTAGAGTGGAAGGTACAGGGCATTTTCTGGATCTGGAGTCGAAACTGGCCGCTGTGAGAGTGCACAGAGCCCTGCTGGAACATCTGCTGAAGCAGCCTGAACCGCAGAGAGCTGAGAGAGCGGCCGGGTTTCACGAGATGGCTATAGGATACGCGTAA |
| *rhlB* | ATGCACGCGATACTGATAGCCATAGGGTCGGCTGGAGACGTGTTTCCTTTTATAGGGCTGGCTAGAACACTGAAACTGAGAGGACACAGAGTGAGTCTGTGCACGATACCTGTGTTTAGAGACGCTGTGGAGCAGCATGGGATAGCGTTTGTGCCGCTGTCGGATGAACTGACATACAGAAGAACGATGGGAGACCCTAGACTGTGGGACCCTAAGACAAGTTTTGGAGTGCTGTGGCAGGCCATAGCTGGTATGATAGAACCTGTGTACGAGTACGTGTCGGCGCAGAGACACGACGATATAGTGGTGGTGGGGAGTCTGTGGGCTCTGGGAGCTAGAATAGCGCATGAAAAATACGGGATACCTTACCTGTCGGCTCAGGTGTCGCCGAGTACACTGCTGAGTGCGCACCTGCCTCCGGTGCATCCTAAGTTTAACGTGCCTGAGCAGATGCCGCTGGCCATGAGAAAACTGCTGTGGAGATGCATAGAAAGATTTAAGCTGGATAGAACGTGCGCTCCTGAGATAAACGCTGTGAGAAGAAAAGTGGGTCTGGAAACACCGGTGAAGAGAATATTTACGCAGTGGATGCACTCGCCTCAGGGGGTGGTGTGCCTGTTTCCGGCCTGGTTTGCTCCTCCGCAGCAGGACTGGCCTCAGCCTCTGCACATGACAGGATTTCCTCTGTTTGATGGAAGTATACCTGGTACGCCGCTGGACGATGAACTGCAGAGATTTCTGGACCAGGGTTCGAGACCGCTGGTGTTTACACAGGGTAGTACGGAGCACCTGCAGGGGGATTTTTACGCGATGGCCCTGAGAGCCCTGGAAAGACTGGGTGCTAGAGGGATATTTCTGACAGGTGCTGGTCAGGAGCCTCTGAGAGGACTGCCTAACCACGTGCTGCAGAGAGCTTACGCGCCTCTGGGTGCTCTGCTGCCTAGTTGCGCTGGACTGGTGCATCCTGGGGGAATAGGAGCTATGAGTCTGGCCCTGGCCGCTGGGGTGCCTCAGGTGCTGCTGCCGTGCGCCCATGACCAGTTTGATAACGCTGAGAGACTTGTGAGACTGGGATGCGGTATGAGACTGGGTGTGCCGCTGAGAGAACAGGAGCTGAGAGGGGCGCTGTGGAGACTGCTGGAGGACCCTGCTATGGCCGCCGCCTGCAGAAGATTTATGGAACTGTCGCAGCCGCACAGTATAGCCTGCGGAAAAGCGGCCCAGGTGGTGGAAAGATGCCATAGAGAGGGTGATGCTAGATGGCTGAAGGCTGCGTCGTAA |
| *rhlY* | ATGAACACAGCCGTGGAACCTTACAAAGCCTCGTCGTTTGACCTGACACACAAACTGACGGTGGAGAAGCACGGGCATACAGCTCTGATAACGATAAACCATCCTCCGGCGAACACATGGGATAGAGACTCGCTGATAGGACTGAGACAGCTGATAGAACACCTGAACAGAGACGATGACATATACGCTCTGGTGGTGACAGGACAGGGTCCTAAATTTTTCTCGGCGGGAGCCGATCTGAACATGTTTGCGGATGGTGACAAGGCTAGAGCGAGAGAAATGGCCAGAAGATTTG  GAGAAGCGTTTGAGGCCCTGAGAGACTTTAGAGGTGTGTCGATAGCCGCTATAAACGGGTACGCTATGGGAGGGGGACTGGAATGCGCCCTGGCTTGCGATATAAGAATAGCGGAAAGACAGGCTCAGATGGCGCTGCCTGAAGCTGCTGTGGGACTGCTGCCTTGCGCTGGGGGAACACAGGCGCTGCCTTGGCTGGTGGGAGAGGGTTGGGCCAAGAGAATGATACTGTGCAACGAAAGAGTGGACGCCGAGACGGCTCTGAGAATAGGTCTGGTGGAACAGGTGGTGGATAGTGGTGAAGCTAGAGGAGCTGCTCTGCTGCTGGCCGCTAAAGTGGCGAGACAGAGTCCTGTGGCCATAAGAACAATAAAGCCGCTGATACAGGGTGCGAGAGAAAGAGCCCCTAACACGTGGCTGCCGGAAGAGAGAGAGAGATTTGTGGATCTGTTTGACGCCCAGGATACGAGAGAAGGGGTGAACGCTTTTCTGGAGAAAAGAGACCCGAAGTGGAGAAACTGCTAA |
| *rhlZ* | ATGAACGTGCTGTTTGAAGAGAGACCTTCGCTGCACGGATTTAGAATAGGTATAGCTACACTGGACGCGGAAAAATCGCTGAACGCCCTGAGTCTGCCGATGATAGAAGCTCTGGCCGCTAAGCTGGACGCTTGGGCGGAGGATGCCGGAATAGCTTGCGTGCTGCTGCGTGGTAACGGGGCCAAAGCCTTTTGCGCCGGGGGAGACGTGAGAAAGCTGGTGGATGCCTGCAGGGAGCAGCCTGGAGAGGTGCCGGCGCTGGCCAGAAGATTTTTCGCGGACGAATACAGACTGGATTACAGAATACACACATACCCTAAACCGTTTATATGCTGGGCCCACGGGTACGTGATGGGTGGGGGAATGGGTCTGATGCAGGGAGCCGGTATAAGAAT  AGTGACGCCTTCGAGTAGACTGGCTATGCCGGAGATAGGGATAGGACTGTACCCTGACGTGGGGGCGTCGTGGTTTCTGGCCAGACTGCCGGGTAGACTGGGGCTGTTTCTGGGACTGAGTGCGGCCCAGATGAACGCGAGAGACGCCCTGGACCTGGATCTGGCCGATAGATTTCTGCTGGACGATCAGCAGGATGCTCTGCTGGCGGGTCTGGTGCAGATGAACTGGAACGAGTCGCCTCAGGTGCAGCTGCACAGTCTGCTGAGAGCTCTGGAACATGAGGCGAGAGGGGAACTGCCTGAGGCTCAGCTGCTGCCTAGAAGACCGAGACTGGACGCTCTGCTGGACCAGCCTGATCTGGCTTCGGCTTGGCAGGCCCTGGTGGCTCTGAGAGACGATGCTGATCCTCTGCTGGCGAGAGGTGCCAAGACACTGGCTGAAGGGTGCCCGATGACGGCGCATCTGGTGTGGCAGCAGATAGAGAGAGCGAGATACCTGTCGCTGGCCGAAGTGTTTAGACTGGAGTACGCTATGAGTCTGAACTGCACAAGACACCCTGACTTTGCCGAAGGAGTGAGAGCTAGACTGATAGACAGAGATAACGCGCCTAACTGGCATTGGCCGCAGGTGGAGAGTATACCGCAGGCCGTGATAGAAGCTCACTTTGAGCCTACATGGGAAGGAGAGCATCCGCTGGCCGGACTGTAA |

**
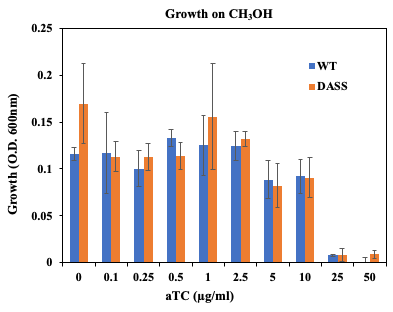
**

**Figure S1.** Effect of anhydro-tetracycline (inducer) concentrations on growth of *M. alcaliphilum* strains WT and DASS at the end of 36h.


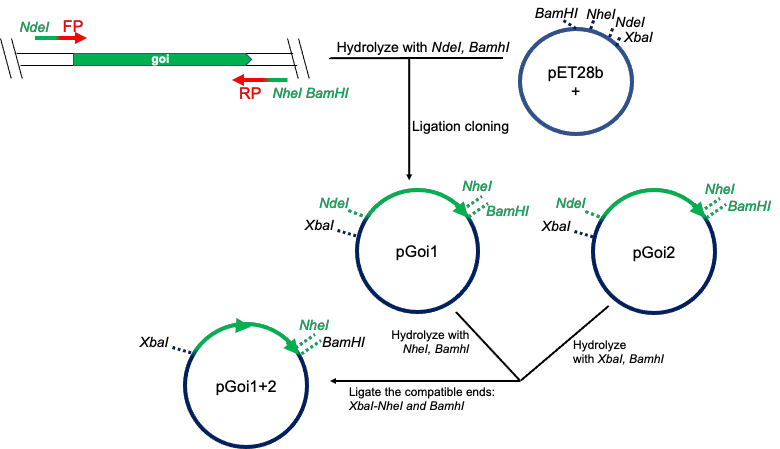


**Figure S2.** Schematic of concatenation assembly of *rhlYZAB* in pET28b(+) vector with individual RBS upstream of each gene. FP and RP, forward and reverse primer; Goi, gene of interest; *BamH1, Nhe1, Nde1, Xba1*, restriction endonucleases.


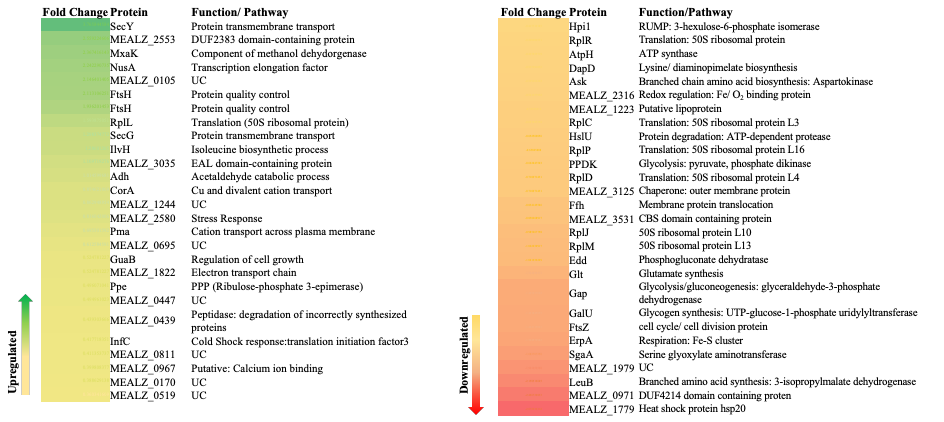


**Figure S3.** Heat map representing the fold change of peptides detected (NSAF, normalized spectral abundance factor) in strain DASS compared to WT at 48h of growth. UC, hypothetical and/or uncharacterized proteins; UC (transmembrane), uncharacterized protein with transmembrane signal peptide domain. Yellow to Green- significant upregulated (p$\leq$0.05 and 3.7$\geq$ FC $\geq$ 0.32); Orange to Red- significant downregulated (p$\leq$0.05 and -0.32$\geq$FC $\geq$ -2.8).

**Figure S4.** Effect of inducer (anhydrotetracycline; aTC) concentrations on growth of *M. alcaliphilum* strain DASS with pCAH01 (empty vector backbone). Black circle-strain DASS; red triangles- strain DASS (pCAH01); dotted line- no inducer added; dashed line- 0.5 µg/ml aTC; solid line- 1 µg/ml aTC.
